# Chemically induced disseminated pythiosis in BALB/c mice: A new experimental model for *Pythium insidiosum* infection

**DOI:** 10.1371/journal.pone.0177868

**Published:** 2017-05-19

**Authors:** Juliana S. M. Tondolo, Érico S. Loreto, Pauline C. Ledur, Francielli P. K. Jesus, Taiara M. Silva, Glaucia D. Kommers, Sydney H. Alves, Janio M. Santurio

**Affiliations:** 1 Departamento de Microbiologia e Parasitologia, Programa de Pós-Graduação em Farmacologia, Centro de Ciências da Saúde, Universidade Federal de Santa Maria, Santa Maria, RS, Brazil; 2 Departamento de Microbiologia e Parasitologia, Programa de Pós-Graduação em Ciências Farmacêuticas, Centro de Ciências da Saúde, Universidade Federal de Santa Maria, Santa Maria, RS, Brazil; 3 Instituto de Química, Programa de Pós-Graduação em Química Orgânica, Laboratório de Processos Tecnológicos e Catálise, Universidade Federal do Rio Grande do Sul, Porto Alegre, RS, Brazil; 4 Departamento de Patologia, Programa de Pós-Graduação em Medicina Veterinária, Centro de Ciências Rurais, Universidade Federal de Santa Maria, Santa Maria, RS, Brazil; 5 Departamento de Patologia, Laboratório de Patologia Veterinária, Centro de Ciências da Saúde, Universidade Federal de Santa Maria, Santa Maria, RS, Brazil; Tallinn University of Technology, ESTONIA

## Abstract

Pythiosis is a severe and life-threatening disease that affects humans and various animal species. We report a model of vascular/disseminated pythiosis occurring after subcutaneous inoculation of 2 x 10^4^ *Pythium insidiosum* zoospores/mL in immunocompromised BALB/c mice. For this model, we carried out two rounds of experiments. First, we evaluated two protocols of immunosuppression before inoculation: cyclophosphamide at 150 mg/kg (CYP group) and cyclophosphamide 200 mg/kg plus hydrocortisone acetate at 250 mg/kg (CYP+HCA group). It was not possible to obtain mortality in the CYP group; however, the combination of CYP+HCA altered disease outcomes, with mortality rates reaching 60%. Second, we used the CYP+HCA immunosuppression protocol to analyze the histological and immunological statuses triggered by disease. When we inoculated immunocompetent mice with *P*. *insidiosum* zoospores, self-healing occurred via increased levels of IL-2, IFN-γ and IL-17A, which are characteristic of the Th1/Th17 cytokine response. For infected and immunosuppressed mice, the cytokine profiles showed high levels of IL-10, IL-6 and TNF-α. Increased IL-10 values are related to fungal infection susceptibility and led us to speculate that infection may be established through suppression of the host immune response. In addition, histopathological evaluation of the kidneys and liver demonstrated the presence of hyphae and the cellular findings suggested an acute vascular inflammation that mimics vascular/disseminated pythiosis in humans. This is the first murine model for pythiosis that is useful both for understanding the pathogenesis of this disease and for evaluating new treatment approaches.

## Introduction

Pythiosis is a chronic pyogranulomatous and life-threatening disease caused by the oomycete *Pythium insidiosum*, which is an oomycetous pathogen that has a microscopic morphology similar to those of true filamentous fungi [1]. This microorganism is unable to synthesize ergosterol because it has an incomplete set of sterol biosynthetic enzymes [2]. This is believed to be the reason behind antifungal treatment failure in pythiosis [1, 3–5].

Infection usually occurs when the host comes in contact with or ingests water contaminated with pathogen zoospores, since *P*. *insidiosum* has an aquatic biological cycle [6]. Most cases occur in tropical and subtropical areas, such as those in Australia, Asia and the Americas, but some cases from temperate areas such as Japan, South Korea, Africa and from some European countries have also been described [1, 3]. Equine is the species most affected by pythiosis, and the Brazilian Pantanal area has the highest incidence and prevalence in the world [7]. In these animals, the disease appears as an ulcerative, pyogranulomatous lesion on cutaneous/subcutaneous tissue [3]. In humans, disease occurs endemically in Thailand where the cutaneous/subcutaneous and disseminated forms are described in approximately 5% and 3% of patients, respectively, whereas ocular (33%) and vascular (59%) pythiosis represent the main forms of the disease [5].

Pythiosis is an aggressive disease that can lead the host to death within a few weeks without therapeutic interventions. In most pythiosis cases, antifungal therapy is ineffective, and surgical removal of the lesion, amputation of the affected limb or enucleation of the affected eye represent the treatment of last resort in humans [8, 9] and animals [10]. However, recurrence rates higher than 40% have already been described, which illustrate the difficulty of controlling this disease [1, 3]. For example, in most cases of vascular pythiosis, the patients undergo affected limb amputation with 40% dying from the disease and 60% surviving with some handicaps [11]. This outcome has been improved using immunotherapeutic approaches in association with antifungals drugs, and surgical cure rates of approximately 55% in humans and 70–80% in horses have been achieved [1]. More recently, antibacterial drugs such as azithromycin, minocycline, linezolid and tigecycline have been described as promising candidate therapies, since these compounds have demonstrated *in vitro* inhibition of the growth of *P*. *insidiosum* isolates [12], *in vivo* curative efficacy against subcutaneous pythiosis in experimental pythiosis [13] and a successful resolution of a presumptive human *Pythium* keratitis [14].

In this context, experimental models are fundamental to understanding pythiosis pathogenesis and evaluating new pythiosis treatments. However, up to now, experimental pythiosis studies on vertebrate animals were conducted with rabbits because they are the only animal species described as susceptible to experimental infection [3, 15]. Although rabbit models are quite useful for the study of this disease, they are more expensive to purchase, their husbandry requires special installations and careful monitoring, and more importantly, there are few immunological and biomolecular reagents available for rabbits [16]. Additionally, this experimental model resulted in only one clinical form of disease, i.e., subcutaneous lesions. In this way, the aim of the present study was to develop a feasible murine model of pythiosis.

## Materials and methods

### Microorganism and zoosporogenesis

The *P*. *insidiosum* isolate (Pi-135), which was obtained from a subcutaneous equine pythiosis infection from southern Brazil, was identified based on its morphological characteristics, as well as with the nucleotide sequence of the ITS1-5.8SrRNA-ITS2 region as previously described by Azevedo et al. [17]. The sequences obtained were analyzed, and nucleotide data for the *P*. *insidiosum* isolate were deposited in GenBank under accession number KJ133548. For the zoosporogenesis induction, first the Pi-135 isolate was grown for 2 days in corn meal agar (CMA, Himedia) supplemented with yeast extract (Himedia) (4 g L^-1^), K_2_HPO_4_ (Vetec) (1.0 g L^-1^) and KH_2_PO_4_ (Vetec) (0.6 g L^-1^) at pH 7.0 ± 0.1 and 37°C. After this primary culture, zoospores were obtained using the zoosporogenesis technique [18] with modifications. Briefly, grass pieces (10 mm) of *Paspalum notatum* were autoclaved (1 atm, 120°C/20 min), layered over the mycelia culture and incubated at 37°C. After 24 h of incubation, the parasitized grass pieces were transferred to a sterile Petri dish containing 20 mL of induction medium (IM) and were incubated for 8–24 h at 37°C. The IM is composed of 0.5 mL solution I (K_2_HPO_4_, 174.18 g L^-1^; KH_2_PO_4_, 136.1 g L^-1^; (NH_4_)_2_HPO_4_ (Vetec), 132.08 g L^-1^; distilled water) plus 0.1 mL solution II (MgCl_2_. 6H_2_O (Vetec), 101.68 g L^-1^; CaCl_2_. 2H_2_O (Vetec), 73.52 g L^-1^; distilled water) dissolved in 1 L distilled water. Zoospore production was observed by inverted microscope, and the cell concentration (cells mL^-1^) was determined using a Neubauer chamber.

### Mice

The mice were purchased from the Bioterium of the Federal University of Pelotas, Brazil, and were employed in the two experiments reported in this study. Fifty female BALB/c mice, 6–8 weeks old, were used (25.30 g ± 2.23). The mice were housed at a temperature of 22°C with 12-h light/dark cycles and provided food and sterile water *ad libitum*. All animal experiments were performed with the approval of the Institutional Animal Care and Use Committee at the Federal University of Santa Maria (Permit number: 071/2014) according to the National Council for Animal Experimentation Control.

### Ethics statement

All standard animal husbandry practices were followed meticulously during the study. Appropriate steps were adopted to keep the mice free from stress or discomfort. To further prevent animal distress, humane endpoints were established at the very beginning of the experiment. Throughout the study, the mice were examined 3–4 times daily for clinical signs, such as rapid or very slow, shallow or labored breathing, weight changes, ruffled fur, hunched posture, impaired ambulation, or lethargy/drowsiness. Other signs taken into consideration included physical and mental alertness, chronic diarrhea and bleeding. These signs were used to decide whether to euthanize the animals during the study or wait until the endpoint, and euthanasia was performed by deepening anesthesia with isoflurane (Cristalia).

### Evaluation of immunosuppression protocol and infection (experiment 1)

To survey the relationship between immunosuppression and survival with infection, we examined two protocols of immunosuppression with 10 mice/group. In the first group, mice received just one intraperitoneal (i.p.) dose of cyclophosphamide (CYP, Baxter, 150 mg/kg) 24 h before infection (CYP group) [19]. The animals in the second group received an i.p. dose of CYP (200 mg/kg) 72 h before the challenge and a subcutaneous (s.c.) dose of hydrocortisone acetate (HCA, Sigma-Aldrich, 250 mg/kg) 24 h before challenge (CYP+HCA group) [20]. On day 0, all mice were infected with a subcutaneous injection of 2 x 10^4^ zoospores/mouse, an inoculum concentration known to induce experimental pythiosis in rabbits [15]. The animals were monitored daily up to the 14^th^ day of infection for signs of morbidity and mortality. The body weights of the animals were recorded daily.

### Immune response and histological analysis (experiment 2)

After evaluating the infection protocols, we chose the CYP+HCA immunosuppression protocol to analyze the histological and immunological status triggered by disease. For this, 20 mice were immunosuppressed with an i.p. dose of CYP (200 mg/kg) and a s.c. dose of HCA (250 mg/kg) at 72 h and 24 h, respectively, before subcutaneous challenge with 2 x 10^4^ zoospores/mouse (PI+CYP+HCA group). As control groups, five non-immunosuppressed and uninfected mice were injected with sterile PBS (Control group), and five non-immunosuppressed mice were inoculated with *P*. *insidiosum* zoospores (PI group) as previously described.

### Histology

Lung, liver and kidney were removed from mice that either died during the course of the infection or were euthanized with isoflurane (inhalation excess). Half of each organ was fixed in 10% buffered formalin for 24 h; after paraffin embedding, 3-μm sections were stained with hematoxylin and eosin (H&E). Immunohistochemistry (IHC) using a polyclonal anti-*P*. *insidiosum* antibody obtained from rabbits was performed as previously described [21] with a modification. Briefly, silanized slides with 3-μm histological sections were used. After dewaxing and tissue rehydration, endogenous peroxidase blockage was performed using 3% hydrogen peroxide followed by antigenic recovery with a TRIS-EDTA solution (pH 9.0) in a microwave oven (maximum power) for 10 minutes. Blocking non-specific reactions was carried out with casein solution (30 minutes at room temperature). As the primary antibody, a rabbit-produced (non-commercial) polyclonal anti-*Pythium insidiosum* antibody, diluted 1: 1000 in PBST and incubated for 60 minutes at 37°C, was used. A peroxidase-polymer (EasyLink One, EasyPath) was used as the detection system, incubated at room temperature for 20 minutes, and labeled by the addition of the chromogen DAB (3–3'-diaminobenzidine tetrachloride, DakoCytomation). The sections were counterstained with Harris hematoxylin, dehydrated, and mounted with synthetic resin (Entellan, Merck) and coverslips. As a positive control, histological sections of a confirmed case of equine pythiosis were used. As negative controls, the same sections were used, and the primary antibody was replaced with PBST. The other organ halves were cultured on CMA at 37°C for one week. If growth occurred, pythiosis was confirmed using the zoosporogenesis technique as previously described under the section “Microorganism and zoosporogenesis” and by a nested PCR technique as previously described [22]. Briefly, the first round of PCR amplification used universal fungal primers (ITS1 and ITS4) to amplify ITS1, 5.8s, and ITS2. During the second PCR round, the obtained fragments were re-amplified using the *P*. *insidiosum*-specific primers PI1 and PI2 [22].

### Immunological studies

For cytokine measurements, the spleens of each mouse from the Control, PI and PI+CYP+HCA groups were aseptically removed, minced and passed through a stainless steel mesh to obtain single cell suspensions in PBS. Thereafter, red blood cells were disrupted in a lysis buffer [8.3 g L^-1^ NH_4_Cl (Vetec), 1 g L^-1^ NaHCO_3_ (Vetec), and 0.04 g L^-1^ EDTA (Vetec)]. The splenocytes were then washed twice with PBS, and the resulting pellet was re-suspended and diluted in a complete tissue culture medium [CTCM: RPMI-1640 (Sigma-Aldrich), 10% fetal bovine serum (Vitrocell Embriolife), and penicillin-streptomycin (100 U and 100 μg mL^-1^, respectively—Sigma-Aldrich)] after the cell viability was assessed by trypan blue (Sigma-Aldrich) exclusion. All lymphocyte suspensions were diluted to 2 x 10^6^ cell mL^-1^ in CTCM. Lymphocytes from all groups were cultivated with RPMI-1640 (non-stimulated cells: N/S) or were stimulated with a *P*. *insidiosum* cell wall β-glucan (Glucan, as a mitogen) at 0.1 mg mL^-1^ [23], and concanavalin A (ConA, Sigma-Aldrich) was used as the mitogen positive control at 0.04 mg mL^-1^.

Supernatant fluids were collected for cytokine determination from splenocyte cultures from each mouse in triplicate after 72 h of culture at 37°C in a 5% humidified CO_2_ atmosphere. The supernatants were then frozen at –80°C until analysis. Cytokines were measured using the mouse Th1/Th2/Th17 cytokine cytometric bead array (CBA) kit (BD Biosciences). The CBA technique was based on seven bead populations, each with a distinct fluorescence intensity, that had been coated with capture antibodies specific for IL2, IL4, IL6, IL10, IL17A, INF-γ and TNF-α proteins. Briefly, 50 μL of each standard or supernatant sample was incubated for 2 hours at room temperature, protected from light, with an equal volume of Phyco Erythrin (detection reagent) and the mixed capture beads. After incubation, 1 mL of wash buffer was added, and the samples were centrifuged at 200 × g for 5 min. The pellet was re-suspended in 300 μL wash buffer and analyzed for cytokine induction. Following the acquisition of sample data (channel FL2-A) on a BD Accuri^™^ C6 flow cytometer (BD Biosciences), the sample results were generated in graphic and tabular format using FCAP array v. 3.0.1 software. Ten standard curves (ranging from 0 to 5,000 pg mL^-1^) were obtained from one set of calibrators, and seven results were obtained from each test sample.

### Statistical analysis

Estimation of survival curves was generated by the Kaplan-Meier method, and the groups were compared by the log-rank test. Cytokine data are expressed as the mean ± S.D, and one-way ANOVA was performed. Comparisons were made versus a respective control group by Holm-Sidak method. Statistical analyses were performed using SigmaPlot software v. 12.5. Differences were considered significant at *p* < 0.05.

## Results

### Immunosuppression protocol and infection (experiment 1)

In the first experiment, the course of infection for the CYP group was less severe than that for the CYP+HCA group, and mortality was not observed. In the CYP group, five mice showed nodular formation at the site of infection, two displayed dry lesions, and three showed no reaction. The loss of corporal weight was not significant (mean ± SD = 23.78 ± 1.21 at day 0 and 23.2 ± 0.97 at day 14). In the CYP+HCA protocol, mortality occurred early during infection, beginning at 48 h after zoospore inoculation in four mice (40%). At 72 h, one mouse died (50%), and the last death was observed 10 days after infection (60%). The calculated mean survival time in the CYP+HCA infected mice was 7.7 days [95% confidence interval (CI), 3.88–11.51; Kaplan-Meier estimate]. Clinical signs such as difficulty breathing, lethargy and/or paralysis of the hind limbs were observed. The remaining mice presented a dry lesion at the site of infection. Body weight loss was higher than in the CYP protocol but did not exceed 20% of initial weight (mean ± SD = 26.40 ± 1.72 at day 0 and 22.6 ± 1.37 at day 14). Once the CYP+HCA protocol allowed the development of a lethal infection, this protocol was used for histological and immunological studies.

### Immune response and histological analysis (experiment 2)

**Fig 1:**
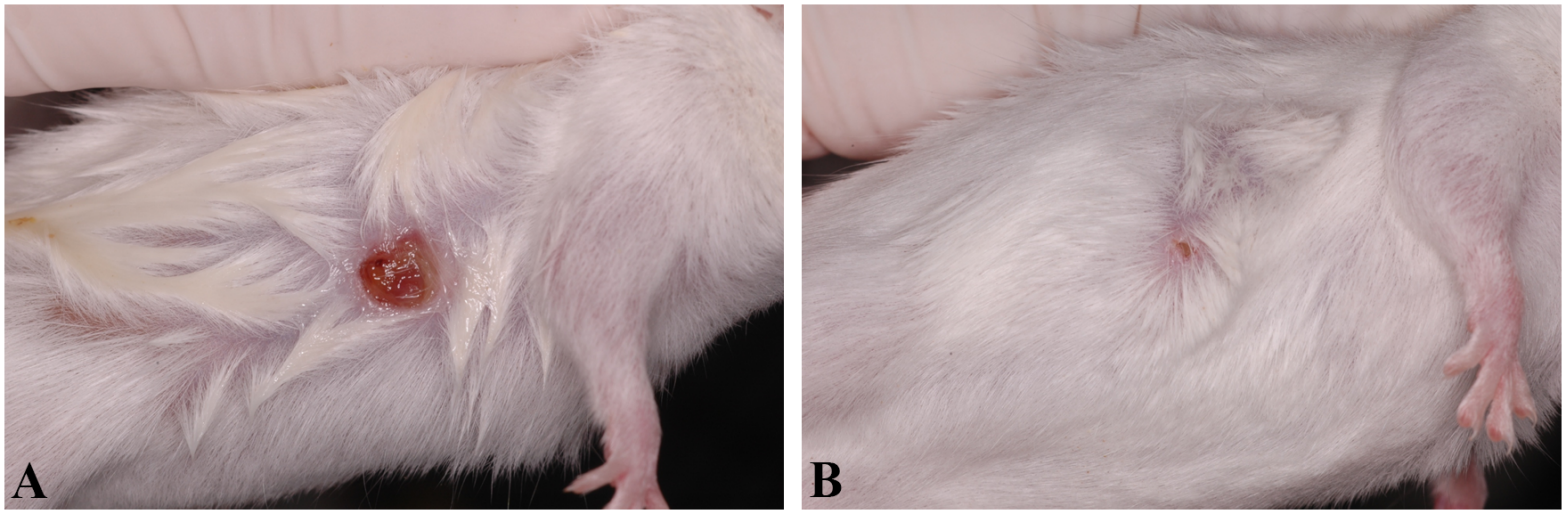
Evolution of subcutaneous lesions of immunocompetent BALB/c mice experimentally infected by the subcutaneous route with 2 x 10^4^ *P*. *insidiosum* zoospores (PI group). A) ulcerative lesions eight days after infection (the lesion was humidified with sterile water for better visualization); B) the same mouse from Fig 1A with spontaneous resolution of the ulcerative lesion 10 days after infection.

**Fig 2:**
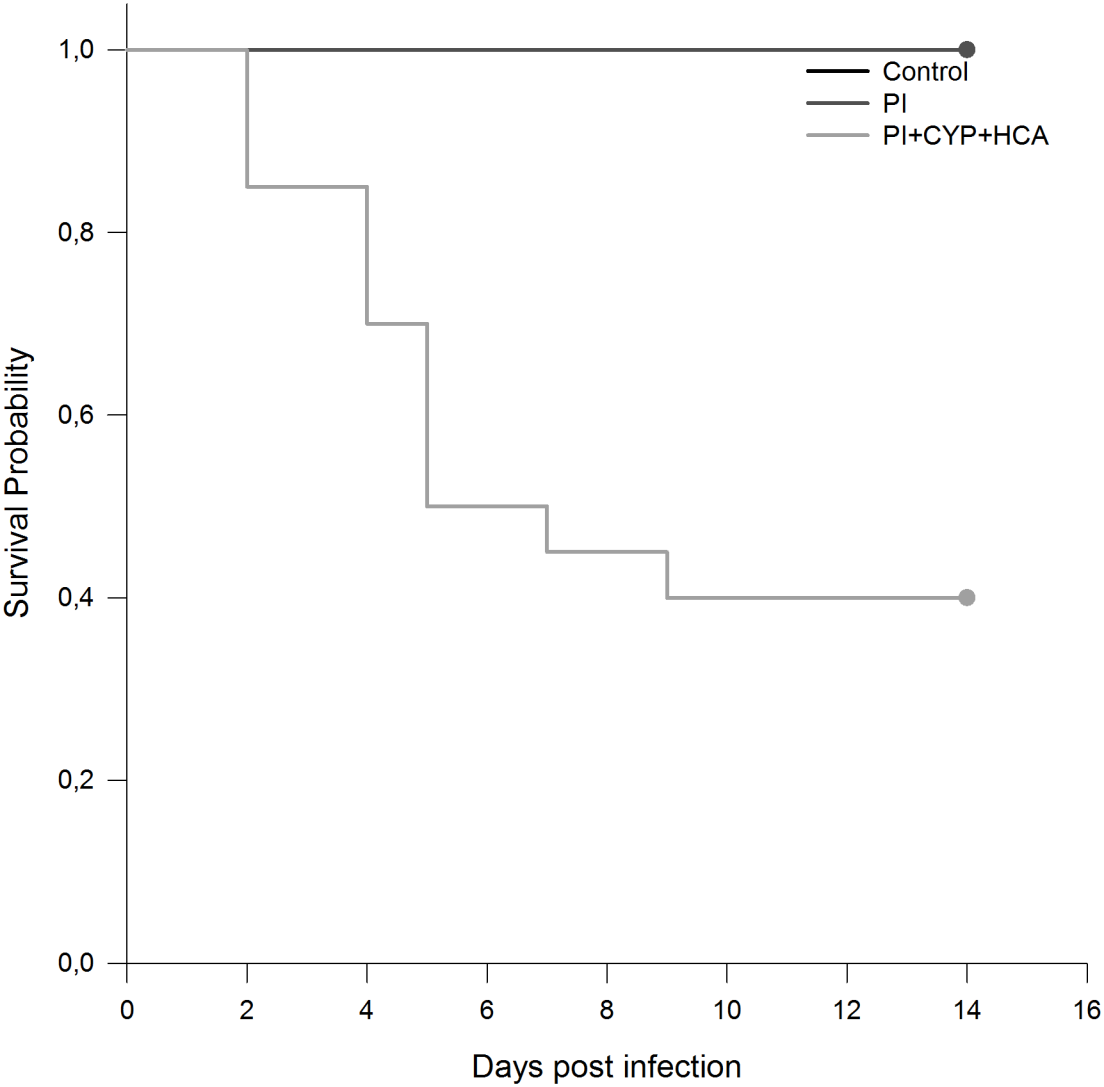
Survival rates of uninfected immunocompetent (Control, *n* = 5), infected immunocompetent (PI, *n* = 5) and immunosuppressed mice with one dose of 200 mg/Kg cyclophosphamide plus one dose of 250 mg/Kg hydrocortisone acetate at 72 h and 24 h, respectively, before infection (PI+CYP+HCA, *n* = 20). Mice were infected by the subcutaneous route with 2 x 10^4^ *P*. *insidiosum* zoospores. The 14-day survival rate was 100% for Control and PI groups and 40.0% for the CYP+HCA group (*p* < 0.001; log-rank test). Control and PI groups are superimposed on the first line.

In the second experiment, we examined the immune responses of uninfected animals as well as immunocompetent and immunosuppressed infected animals. In the PI group, on the 7th day after inoculation, subcutaneous nodules were observed in 4/10 (40%) mice. Between days 7 and 8, the lesions ulcerated (Fig 1A), but spontaneous remission could be observed in all animals after the 10th day of inoculation (Fig 1B). The mortality rates in the PI+CYP+HCA group reached 60% and occurred early during infection, as well as observed during the evaluation of immunosuppression and infection protocols. Mortality began with three mice at day 2 (15%), three mice at day 4 (30%), four mice at day 5 (50%), and one mouse at day 7 (55%). The last deaths occurred at day 9 (60%) post-infection (Fig 2). The loss of corporal weight was not significant (mean ± SD = 23.78 ± 1.21 at day 0 and 23.2 ± 0.97 at day 14). Mice presented with difficulty breathing, lethargy and/or paralysis of the hind limbs. The calculated mean of survival in PI+CYP+HCA group was 8.3 days (CI, 6.05–10.54; Kaplan-Meier estimate).

### Histology

**Fig 3:**
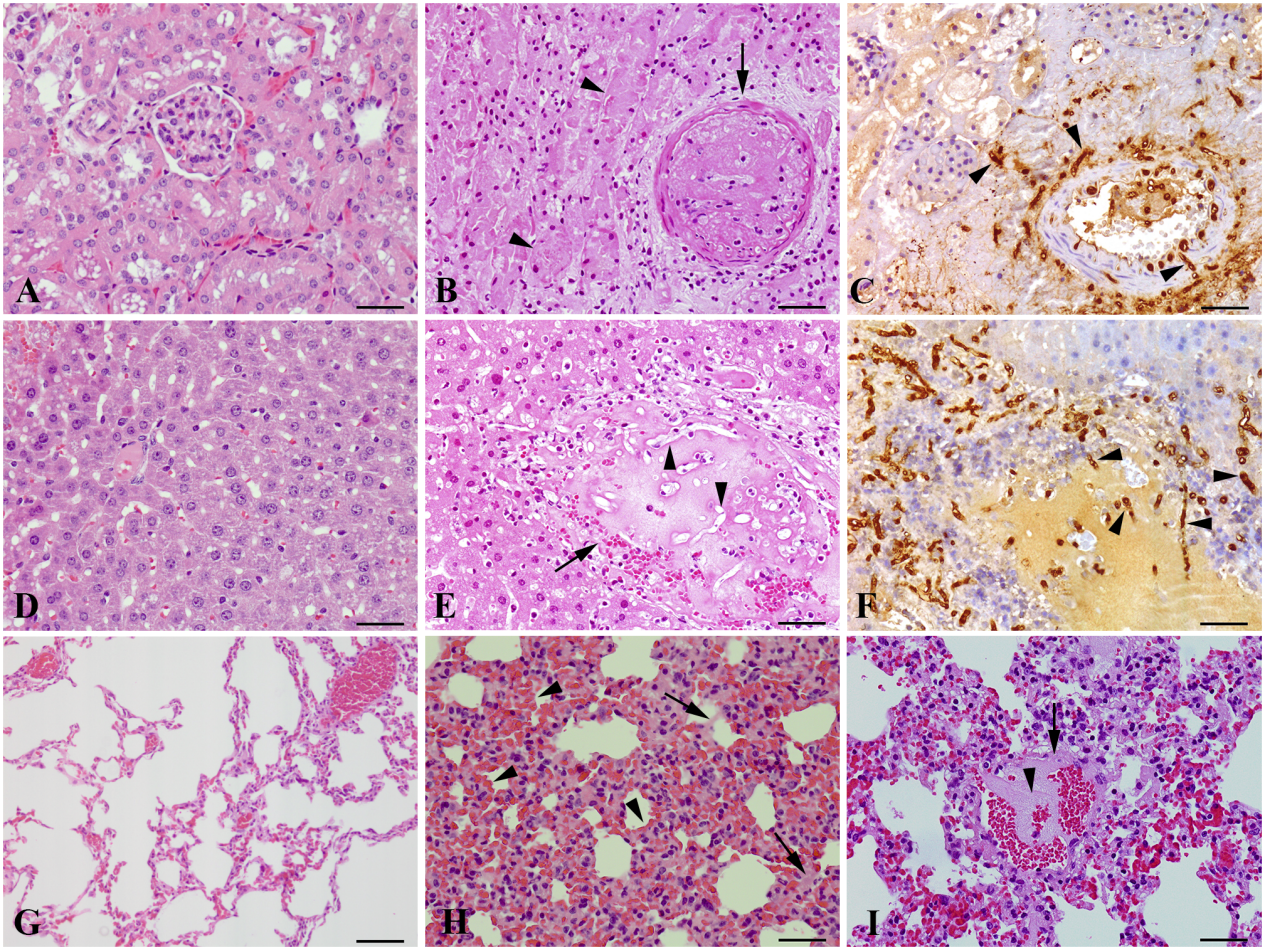
Histological lesions and immunohistochemistry of pythiosis in the kidneys, liver and lungs of BALB/c mice from the immunosuppressed (PI+CYP+HCA) group that was subcutaneously infected with *P*. *insidiosum*. A) Histological sections of kidney from a healthy control mouse (hematoxylin-eosin [HE, Bar = 20μm]). B) Kidney sections from the infected mouse with thrombosis (arrow) and tubular necrosis (arrowheads) (HE, Bar = 20μm). C) Positively stained hyphae are observed in the arterial small thrombus and vessel wall and surrounding the kidney (arrowheads) (immunohistochemistry [IHC] anti-*P*. *insidiosum*, peroxidase-polymer method, Bar = 20 μm). D) Histological sections of liver from a healthy control mouse (HE, Bar = 20 μm). E) Liver of the infected mouse with thrombus (arrow) containing negative profiles of hyphae (arrowheads) (HE, Bar = 20 μm). F) Positively stained hyphae are observed in the arterial thrombus and vessel wall and surrounding the liver (arrowheads) (IHC anti-*P*. *insidiosum*, Bar = 20 μm). G) Histological sections of the lung from a healthy control mouse (HE, Bar = 50 μm). H) Lung with severe congestion in septal capillaries and multifocal hemorrhage (arrowhead) in alveoli with mild edema (arrow) and fibrin (HE Bar = 20 μm). I) Lung with fibrinoid necrosis (arrow) and thrombosis (arrowhead) in one arteriole (HE, Bar = 20 μm).

Fig 3(A) to 3(I) demonstrates the main histological lesions and immunohistochemistry of pythiosis in the kidneys, liver and lungs of BALB/c mice from the immunosuppressed (PI+CYP+HCA) group that was subcutaneously infected with *P*. *insidiosum*. Focally extensive coagulation necrosis areas associated with severe multifocal thrombosis in arteries, arterioles and venules were similarly evident in the kidneys and liver (Fig 3B and 3E). Neutrophilic vasculitis was observed, and neutrophils were numerous among the thrombi. Negative hyphae profiles were observed in the midst of thrombi and in the vessel walls (Fig 3E). The hyphae were branched and occasionally septate, and some were observed in the interstitium of the necrotic areas. They stained positively for *P*. *insidiosum* by immunohistochemistry. Lung histopathology showed severe congestion in the septal capillaries and multifocal hemorrhage in the alveoli with the presence of fibrin and mild edema (Fig 3H). There was also mild to moderate fibrinoid necrosis in the arteriole walls, sometimes with mild thrombosis (Fig 3I), but hyphae were not observed.

### Immune response

**Fig 4:**
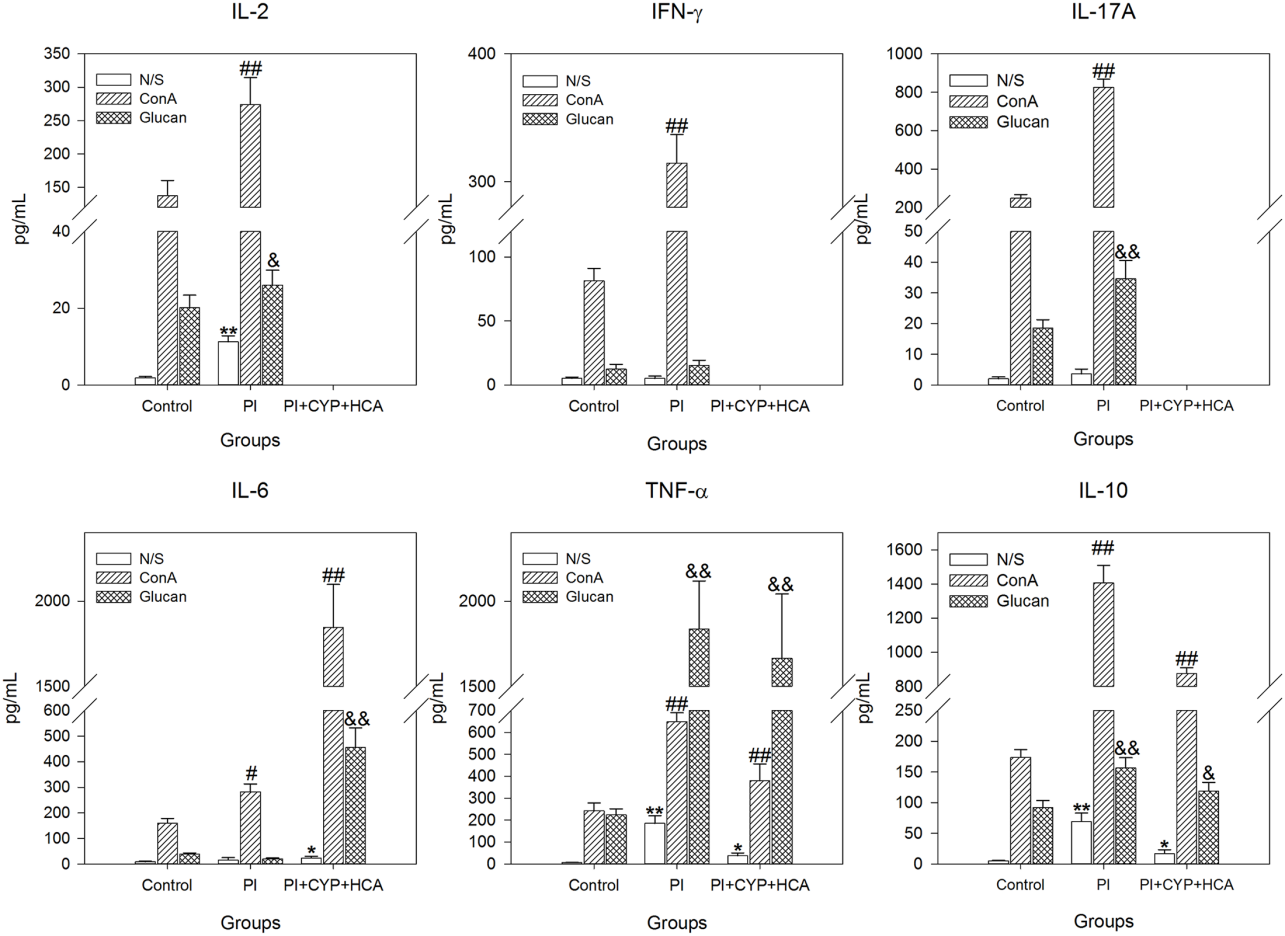
Cytokine secretion by uninfected immunocompetent (Control), infected immunocompetent (PI) and infected immunosuppressed (PI+CYP+HCA) mice. Mouse spleen cells were cultured in the presence of β-glucan at 0.1 mg mL^-1^ (Glucan), concanavalin A at 0.04 mg mL^-1^ (ConA) or RPMI-1640 (not stimulated, N/S) and incubated for up to 72 h in 5% CO_2_ at 37°C. Cytokine levels in lymphocyte culture supernatants were measured by flow cytometry. Infection was established with a subcutaneous injection of 2 x 10^4^ *P*. *insidiosum* zoospores. The Control group received injections of only phosphate-buffered saline (PBS). The data are presented as the means ± SD. An * indicates significant difference compared with the N/S control group (**p < 0.001; *p < 0.05). # indicates significant difference compared with the ConA control group (## *p* < 0.001; # *p* < 0.05). & indicates significant difference compared with the Glucan control group (&& *p* < 0.001; & *p* < 0.05).

To determine whether *in vivo* experimental pythiosis could modify cytokine production, we analyzed the supernatants of cultured spleen cells. The results of cytokines levels from cultures of non-stimulated (N/S), β-glucan-stimulated (Glucan) and ConA-stimulated (ConA) spleen cells from infected and uninfected mice are described in Fig 4. For spleen cells from the N/S PI group, significant increases were observed in IL-2, TNF-α and IL-10 production when compared with the N/S control. In the ConA PI group, IL-2, IFN-γ, IL-17A, IL-6, TNF-α and IL-10 were higher than in the ConA control group. In the Glucan PI group, IL-2, IL-17A, TNF-α and IL-10 levels were significantly increased compared with those in the Glucan control. IL-4 was not detected in the PI group. For N/S, ConA and Glucan cells from PI+CYP+HCA group had significantly higher levels of IL-6, TNF-α and IL-10 than did the N/S, ConA and Glucan control groups, respectively. IL-2, IFN-γ, IL-17A and IL-4 levels were not detected in the PI+CYP+HCA group.

## Discussion

Several studies evaluating fungal infections in mice use chemical immunosuppression protocols to induce disease [24]. CYP is a non-phase specific cytotoxic agent that can inhibit both humoral and cellular immunity, which affects granulocyte and lymphocyte activities [25]. The effects of corticosteroids are pleiotropic, affecting both T and B cells, macrophages, granulocytes, and monocytes [26]. The combination of cytotoxic chemotherapy and corticosteroid therapy appears to be additive and influence the outcome of experimental infections, since these drugs alone may not result in consistent lethal infections [27]. In this way, we induced pythiosis in mice immunosuppressed with a combination of CYP and HCA, which is a well-documented protocol in experimental invasive pulmonary aspergillosis (IPA) [20, 26, 28, 29].

Immunosuppression with the CYP+HCA protocol greatly altered disease outcomes, with subcutaneous zoospore inoculation leading to death, whereas immunocompetent mice developed a self-healing ulcerative subcutaneous lesion. Mortality rates reached 60%, and during the experiment, we observed that mice sometimes had difficulty breathing, lethargy and/or hind limb paralysis. Animal death can be explained by the dissemination of the oomycete and the colonization of vital organs, probably caused by impairment of humoral and cellular host responses. For many years, there was a consensus that experimental pythiosis could be reproduced only in rabbits [3]. However, Zanette et al. recently described that an experimental model of pythiosis can be reproduced in Toll-deficient *Drosophila melanogaster* [30]. In this context, this is the first study to propose a murine model of pythiosis.

In our experiments, pythiosis was characterized by histopathology and tissue culture. The histopathological findings in the kidneys and livers suggested an acute vascular inflammation, and these results in combination with hind limb paralysis mimic the vascular and disseminated forms of the disease in untreated human patients [5]. The invasion of large arteries of the limbs is commonly diagnosed in human patients from Thailand. Arterial pythiosis is a life-threatening condition, especially when *P*. *insidiosum* reaches the aorta and when the major cause of death is the rupture of an aortic aneurysm [5]. In this way, our murine pythiosis infection model can be very useful to study therapeutic approaches in human pythiosis.

The host immune response to natural *P*. *insidiosum* infection is thought to be a Th2 response with eosinophilic inflammation and the release of IL-4 and IL-5 associated with disease progression [1]. Indeed, in a human patient with vascular pythiosis, high levels of IL-4 and IL-5 cytokines have been detected during infection [31]. However, evaluation of the cytokine levels in animals with pythiosis had not been performed.

We observed that immunocompetent mice could achieve self-healing by a typical Th1/Th17 response, with an increase in the production of IFN-γ and IL-17A. Indeed, many fungal infections are predicted to be cured when a Th1 and/or Th17 response is established [32, 33]. On the other hand, infected immunosuppressed animals had abrogated IFN-γ and IL-17A levels and presented high levels of IL-6, IL-10 and TNF-α compared with the uninfected immunocompetent mice. This last cytokine profile, with up-regulation of IL-10, but not a typical Th1 response with high levels of IFN-γ, may be partially responsible for the impediment of total clearance of the agent. IL-10 represents an important cytokine that may affect the Th1/Th2 balance in fungal infections. This cytokine is known to be a major immunoregulatory cytokine influencing the development and the production of Th cells and numerous pro-inflammatory cytokines [34]. High levels of IL-10 that downregulate IFN-γ production are detected in chronic candidiasis, in the severe forms of endemic mycoses and in neutropenic patients with aspergillosis, and thus have been linked to susceptibility to fungal infections [35, 36].

Our data are in agreement with previous studies of the pathogenesis of IPA. In these studies, chemotherapy-treated mice presented high levels of pro- and anti-inflammatory cytokine responses (TNF-α and IL-10, respectively), and IL-10 was associated with an increase in mortality and pathogenesis of IPA [26, 29]. Because several cell types produce IL-10, mainly T regulatory cells, early secretion of this cytokine cannot be used as a marker of Th2 activation [37]. Furthermore, the absence of IL-4 secretion observed during our study renders it difficult to attribute infection development to a typical activation of Th2 cells.

A recent study with the oomycete *Saprolegnia parasitica* demonstrated a strong induction of pro-inflammatory cytokines triggered by the cell wall components of this microorganism. Furthermore, severe suppression of the gene expression related to adaptive immunity through downregulation of T-helper cell cytokines demonstrated a Th1/Th2 imbalance in infected animals. They concluded that an active suppression of fish adaptive immunity during infection was responsible by immune system failure to respond adequately to infection [38].

In the present work, we have successfully developed, for the first time, a new model of *P*. *insidiosum* infection in immunosuppressed mice using a combination of CYP+HCA drugs. This model enables research and future studies to explore unanswered questions concerning the immunopathogenesis and treatment of pythiosis in an acceptable animal system that mimics vascular/disseminated disease in humans. We concluded, based on cytokine profiles and histopathological analysis, that immunosuppression is critical to development of pythiosis in mice, and we propose that the immunosuppressed-infected mice is a simple, reproducible and reliable model that can be used to study this disease. These results should be carefully interpreted, and further studies, using geographically and genetically diverse *P*. *insidiosum* strains, are needed to determine the reproducibility of this model.
